# Case report: Primary cardiac undifferentiated sarcoma complicated by esophagus stenosis

**DOI:** 10.3389/fonc.2024.1530403

**Published:** 2025-01-21

**Authors:** Yuxin Ge, Xiaopan Lv, Rui Zhang, Dongxiao Hao, Guifei Si, Yuquan Li, Xuemin Yuan, Xiuping Li

**Affiliations:** ^1^ School of Clinical Medicine, Shandong Second Medical University, Weifang, Shandong, China; ^2^ Department of Gastroenterology, Linyi People’s Hospital, Linyi, Shandong, China

**Keywords:** cardiac undifferentiated sarcoma, endoscopy, esophagus stenosis, immunohistochemistry, pathology

## Abstract

In this study, we present the case of a 38-year-old woman who was diagnosed with primary cardiac undifferentiated sarcoma after hospital admission. Following postoperative treatment that included radiotherapy and immunotherapy, the patient developed esophagus stenosis.

## Introduction

Primary cardiac tumors are rare with an incidence between 0.0017% and 0.28% (1). Twenty-five percent of cardiac tumors are malignant, and nearly 20% of these are sarcomas. Among these, Cardiac angiosarcomas constitute 37% of cardiac sarcomas, while undifferentiated sarcomas account for less than 24%, leiomyosarcomas constitute 8% to 9% of cardiac sarcomas, and rhabdomyosarcomas account for 4% to 7%. In addition, Osteosarcoma, fibrosarcoma, liposarcoma, and synovial sarcoma represent a small proportion of cardiac sarcomas (2, 3). Cardiac sarcomas are often asymptomatic until advanced, and even then produce non-specific symptoms such as palpitations, shortness of breath, dyspnea, chest pain, syncope, anemia, and weight loss (4–6). As a result, they can easily be misdiagnosed as other conditions, such as pneumonia or pericarditis (4). The dismal prognosis results from extensive local invasion or distant metastases at presentation. The initial diagnosis of cardiac sarcoma primarily relies on imaging techniques such as echocardiography, MRI and PET-CT, although a definitive histopathological diagnosis is essential (7, 8). In this report, we described a case that underwent initial cardiac tumor resection, and was confirmed to be undifferentiated sarcoma based on pathological findings. Following postoperative treatment that included radiotherapy and immunotherapy, the patient developed esophagus stenosis. To the best of our knowledge, this is the first case report detailing a primary cardiac undifferentiated sarcoma complicated by esophagus stenosis.

## Case reports

**Figure 1:**
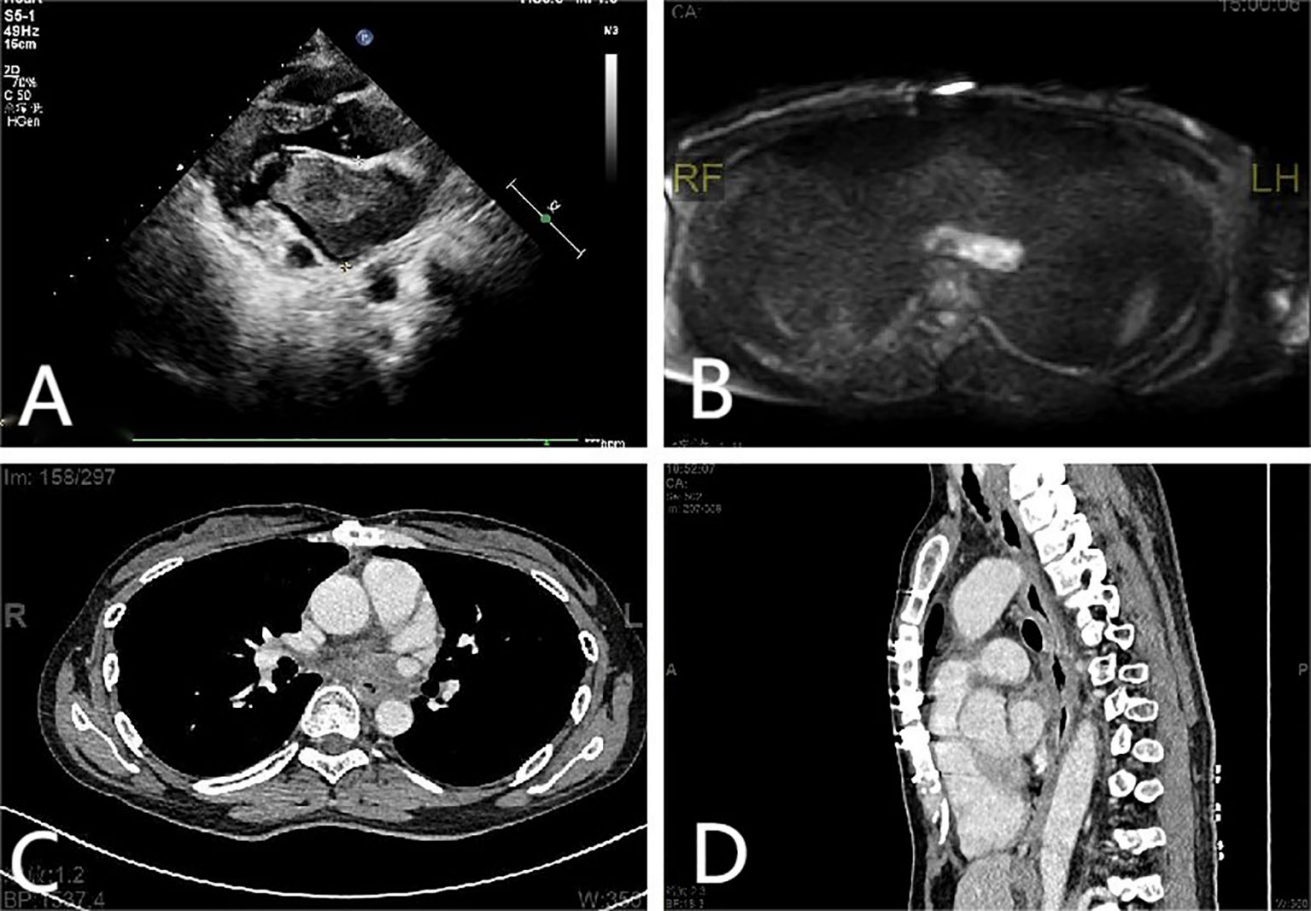
**(A)** Echocardiographic findings: A mass measuring approximately 69 mm × 41 mm was identified in the anterior wall of the left atrium. **(B)** Cardiac MRI scan findings: abnormal signals in the posterior wall of the left atrium. **(C, D)** Mediastinal CT scan findings: the posterior wall of the left atrium and the posterior esophagus were thickened. The gap between the esophagus and the left atrium disappeared, and tumor cell infiltration of the esophagus was considered.

**Figure 2:**
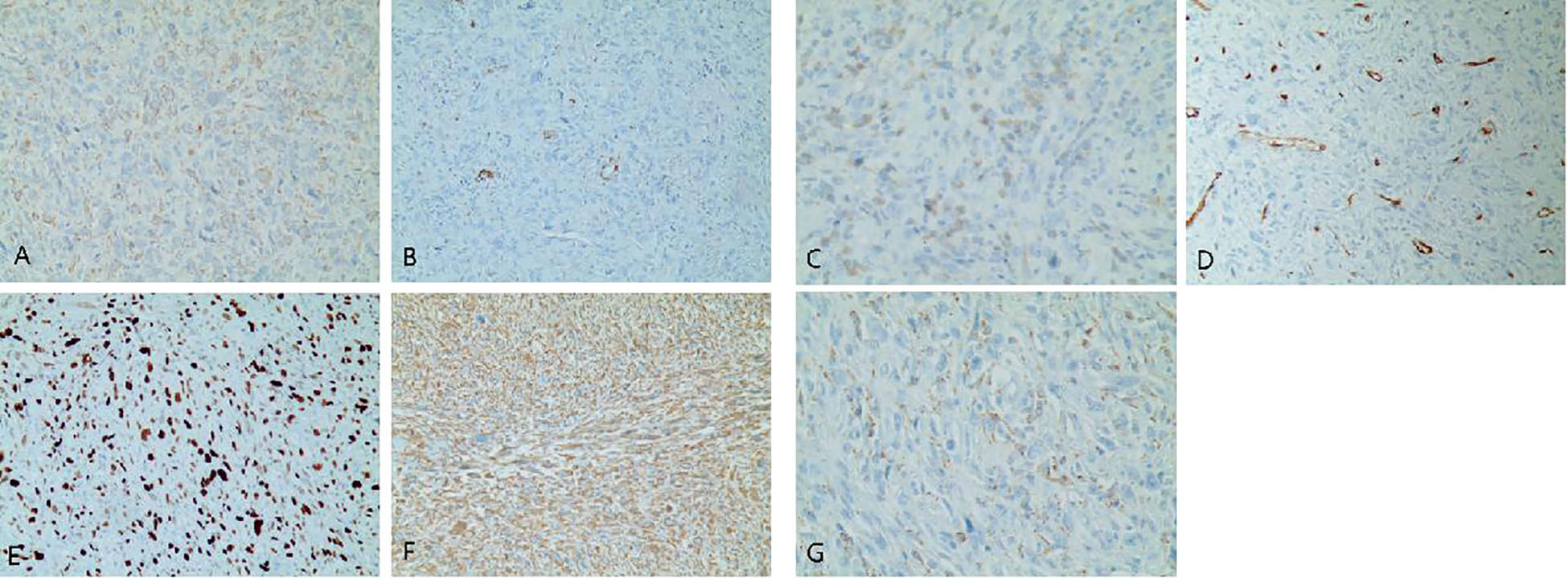
**(A–G)** Immunohistochemistry of the left atrium mass. **(A)** CK (focal +). **(B)** SMA (focal +). **(C)** Desmin (focal +). **(D)** CD34 (vascular +). **(E)** Ki67-MIB1 (50%). **(F)** Vimentin (+). **(G)** CD68 (+).

**Figure 3:**
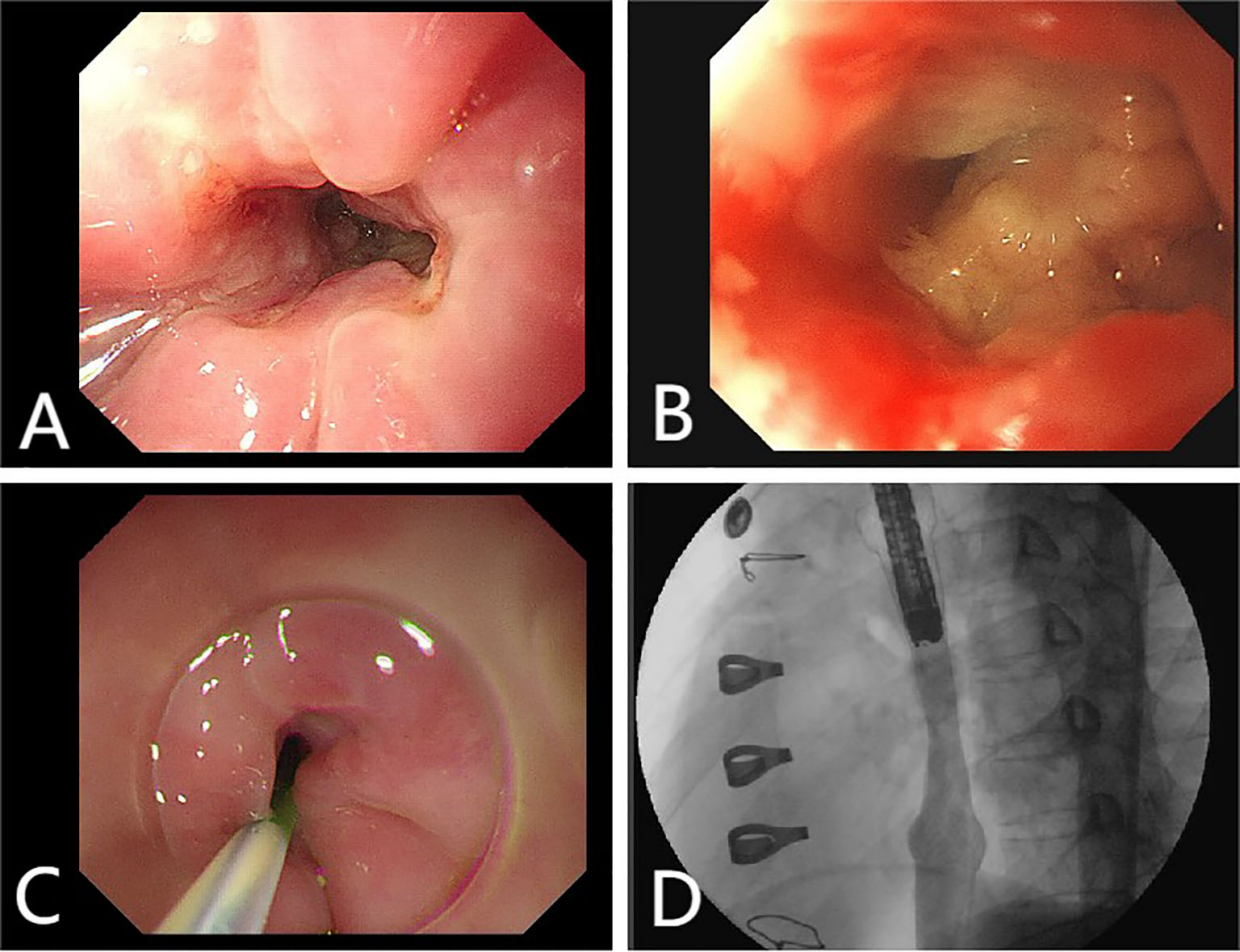
**(A)** Endoscopy reveals an esophagus ulcer covered with white exudate, narrowing of the lumen. **(B)** Endoscopy reveals an esophagus ulcer. **(C, D)** Esophagus stent implantation.

**Figure 4:**
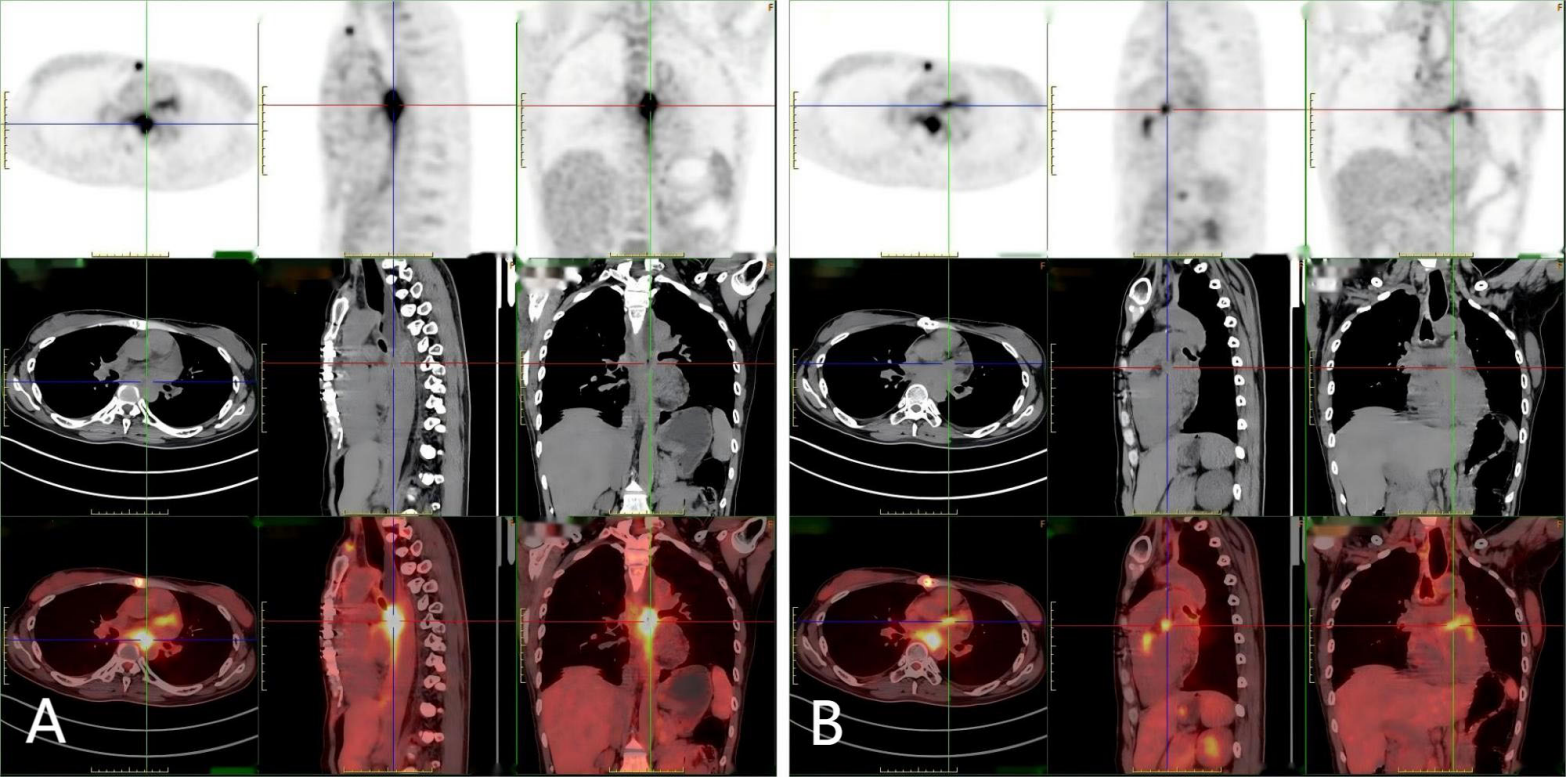
**(A, B)** A ^18^F-FDG-PET-CT scan: Hypermetabolic lesion in the left atrium wall and esophagus.

A 38-year-old female patient was admitted to the hospital on December 9, 2023 with complaints of “palpitations for 1 week.” She did not report any significant chest tightness, wheezing, or other noteworthy symptoms. She was healthy in the past and had no family history of hereditary disease. An outpatient echocardiogram revealed a large left atrium with a medium to strong echo mass (69 mm x 41 mm) attached to the roof of the left atrium. The mass had an irregular shape with uneven internal echogenicity and was partially protruding into the left ventricle near the mitral valve (**Figure 1A**). Cardiac mass resection was performed on December 12, 2023. The right atrium and interatrial septum were incised, revealing a large tumor (7 cm x 5 cm x 5 cm), and the tumor is somewhat resilient in texture (such as palpating the nose tip), that occupied the left atrium and extended into the openings of the left and right pulmonary veins. The tumor was completely excised along its attachment to the surrounding tissues. The postoperative immunohistochemical analysis revealed the following results: CK (focal +), SMA (focal +), Desmin (focal +), S-100 (–), SOX 10 (–), CD34 (vascular +), Ki67-MIB1 (50%), Vimentin (+), MyoD1 (–), Myogenin (–), CD68 (+). (**Figures 2A–G**), confirmed that the tumor was consistent with cardiac sarcoma, undifferentiated sarcoma. The patient’s recovery following surgery was uneventful. However, on January 25, 2024, cardiac MRI revealed abnormal signals in the posterior wall of the left atrium, raising concerns of tumor recurrence (**Figure 1B**). Radiotherapy was started in February, 2024, (18 times, Radiation dose: 3x18Gy) and was completed on March 1, 2024. In accordance with the guidelines from the Chinese Society of Clinical Oncology for the Diagnosis and Treatment of Soft Tissue Sarcoma, the patient began oral anlotinib (10 mg QD) during radiotherapy. Regular immunotherapy with cindarizumab (administered once every 21 days) commenced on February 28, 2024. In April 2024, the patient was readmitted due to dysphagia. Gastroscopy indicated that the distance between the esophagus and the incisor was approximately 28 cm, revealing an esophagus ulcer covered with white exudate, narrowing of the lumen, and obstruction preventing endoscopic passage (**Figure 3A**). Mediastinal CT showed that after left atrial surgery, the posterior wall of the left atrium and the posterior esophagus were thickened (**Figures 1C, D**). PET-CT revealed thickening of the left atrial wall and the esophagus wall, with increased FDG metabolism (**Figures 4A, B**). On August 8, 2024, follow-up gastroscopy demonstrated an esophagus ulcer located about 30 cm from the incisor, consistent with an inability to pass the endoscope (**Figure 3B**). The comprehensive analysis considered the patient’s recurrence of cardiac undifferentiated sarcoma and invasion of the esophagus, resulting in esophagus stenosis. After consulting with the cardiovascular surgeon, the patient was informed about the necessity and risks of undergoing another surgical procedure. However, the patient and his family ultimately refused further surgical treatment. For treatment, a multidisciplinary team, including oncologists and gastroenterologists, recommended esophagus stent implantation, which was successfully performed on August 28, 2024 (**Figures 3C, D**). The procedure was uneventful, and the patient’s swallowing capability was deemed acceptable. As of the time of writing, the patient remains alive and independent.

## Discussion

The incidence of primary cardiac tumors ranges from 0.0017% to 0.28% (1). These sarcomas typically have a poor prognosis, as they are often widely disseminated and prone to hematogenous metastasis to the lungs or liver by the time of diagnosis (9, 10). Undifferentiated sarcoma, the most aggressive type of cardiac malignant tumor, accounts for approximately 12% of all primary cardiac sarcomas and is associated with an extremely poor prognosis (11). Due to their local aggressiveness, complete resection of undifferentiated sarcomas is often not feasible, with only about 12% of patients achieving R0 resection (complete response) (12). Despite aggressive treatment, the median overall survival for patients with cardiac sarcomas is low, typically ranging from 6 to 12 months (13). Notably, patients who receive multimodal therapy may experience better survival outcomes compared to those who undergo surgery alone (14). The treatment of undifferentiated sarcoma necessitates a multidisciplinary approach, often derived from case reports and series, due to a lack of robust data, randomized clinical trials, or standardized treatment protocols (15, 16). In this case, the patient was pathologically confirmed as undifferentiated sarcoma after cardiac mass resection, and active intervention was immediately taken, including radiotherapy, immunization and targeted therapy. Currently, the patient is experiencing esophagus stenosis, ^18^FDG-PET-CT showed a hypermetabolic lesion in the left atrium wall and esophagus, so we considered the possibility of recurrence and invasion of cardiac malignant tumors (17). However, pathological results did not confirm any malignancy, which may be attributed to the effects of prior radiotherapy. Research indicates that the incidence of esophagus stenosis is 1%-2% when the radiation dose is less than 60 Gy, increasing to 5%-6% when the radiation dose exceeds 60 Gy (18). Studies have found that there is a significant correlation between radiotherapy dose and the occurrence of esophagus stenosis. As the radiation dose increases, the degree of fibrosis in the esophageal muscle layer and submucosa progressively worsens, thereby elevating the risk of esophageal stenosis (19, 20). Therefore, the specific causes need to be further examined. Finally, the patient underwent esophagus stent implantation. The procedure was successful, and the patient reported improved swallowing function postoperatively. From the review of literature, we conclude that although surgical intervention was necessary for resection of the tumor, it is important to identify malignant tumors early, in order to enable patients to undergo radiotherapy or chemotherapy as soon as possible. In addition, We believe that cardiac MRI examinations should be enhanced prior to surgery to provide better guidance during the procedure. In this case, the cardiac MRI was not adequately improved before the operation, which reflects our oversight. In the future, we should adopt a more rigorous approach to diagnosis and treatment, ensuring that preoperative examination and evaluation are thoroughly optimized. The treatment of undifferentiated cardiac sarcoma requires a multidisciplinary approach. Advances in anti-tumor immunotherapy, targeted therapy, and chemotherapy offer new options for managing cardiac sarcomas. Moving forward, more basic and clinical research is needed to develop additional treatment strategies for this disease, ultimately aiming to extend patient survival.

## Data Availability

The original contributions presented in the study are included in the article/supplementary material. Further inquiries can be directed to the corresponding author.
